# Impact of Community-Based Larviciding on the Prevalence of Malaria Infection in Dar es Salaam, Tanzania

**DOI:** 10.1371/journal.pone.0071638

**Published:** 2013-08-14

**Authors:** Mathieu Maheu-Giroux, Marcia C. Castro

**Affiliations:** Department of Global Health and Population, Harvard School of Public Health, Boston, Massachusetts, United States of America; Burnet Institute, Australia

## Abstract

**Background:**

The use of larval source management is not prioritized by contemporary malaria control programs in sub-Saharan Africa despite historical success. Larviciding, in particular, could be effective in urban areas where transmission is focal and accessibility to *Anopheles* breeding habitats is generally easier than in rural settings. The objective of this study is to assess the effectiveness of a community-based microbial larviciding intervention to reduce the prevalence of malaria infection in Dar es Salaam, United Republic of Tanzania.

**Methods and Findings:**

Larviciding was implemented in 3 out of 15 targeted wards of Dar es Salaam in 2006 after two years of baseline data collection. This intervention was subsequently scaled up to 9 wards a year later, and to all 15 targeted wards in 2008. Continuous randomized cluster sampling of malaria prevalence and socio-demographic characteristics was carried out during 6 survey rounds (2004–2008), which included both cross-sectional and longitudinal data (N = 64,537). Bayesian random effects logistic regression models were used to quantify the effect of the intervention on malaria prevalence at the individual level. Effect size estimates suggest a significant protective effect of the larviciding intervention. After adjustment for confounders, the odds of individuals living in areas treated with larviciding being infected with malaria were 21% lower (Odds Ratio = 0.79; 95% Credible Intervals: 0.66–0.93) than those who lived in areas not treated. The larviciding intervention was most effective during dry seasons and had synergistic effects with other protective measures such as use of insecticide-treated bed nets and house proofing (i.e., complete ceiling or window screens).

**Conclusion:**

A large-scale community-based larviciding intervention significantly reduced the prevalence of malaria infection in urban Dar es Salaam.

## Introduction

The Ross-Macdonald model of malaria transmission suggests that control methods that reduce adult mosquitoes’ longevity can achieve greater malaria reduction than strategies that target larval stages. Yet, Larval Source Management (LSM), such as the use of larvicides and the draining of breeding habitats, has historically been a very successful tool to reduce mosquito density [1] – examples include the elimination of *Anopheles arabiensis* from Egypt [2] and Brazil [3], malaria control in the Zambian copperbelt (1930–1950) [4], Dr. Gorga’s work during the construction of the Panama canal [5], and the vector control program of the Tennessee Valley Authority [6]. With the discovery of DDT, however, such approaches where disfavored as exemplified by the almost exclusive use of this potent insecticide during the Global Malaria Eradication Program (1955–1969) [7]. In addition, LSM programs were often associated with vertical, authoritarian management. Currently, there are few examples of LSM initiatives in post-colonial Africa [8]–[10]. LSM is often perceived as a secondary malaria control strategy, labor-intensive, requiring strong managerial support and oversight for monitoring and evaluation [11], [12], and often beyond the financial and operational capabilities of most malaria endemic areas in sub-Saharan Africa [13].

Such considerations might explain the insufficient evidence-base of LSM in post-colonial Africa, and the contemporary prioritization of malaria control programs that rely on Insecticide-Treated Nets (ITNs) and Insecticide Residual Spraying (IRS) as the main vector control measures. Nevertheless, a renewal of interest in applications of LSM within the sub-Saharan context has been observed recently [14]–[18]. In fact, in April of 2012, the World Health Organization (WHO) released an interim position statement [19] on the use of larvicides for malaria control in sub-Saharan Africa, recognizing that larviciding should be considered for malaria control but only in areas where breeding sites are ‘*few, fixed and findable*’ [19]. Larval control is regarded as being of secondary importance in comparison with IRS and ITNs. Although the WHO acknowledges that larvicides could be effective as one of the leading methods of vector control in urban areas of sub-Saharan Africa, it highlights the lack of recent and sound evidence of its effectiveness. Few contemporary studies have assessed the effectiveness of larvicides on malaria infection. Studies in highland valley communities of Kenya [20] and urban Tanzania [21] demonstrated substantial reduction in malaria prevalence, while no reductions were observed in a study conducted in a rural setting in The Gambia [22]. Strong empirical evidence on the causal effect of larviciding on malaria infection is difficult to obtain since larviciding interventions need to be implemented and scaled-up over large areas, appropriate control groups with similar malaria ecology are difficult to find, and the cost of such trials can be prohibitively expensive [14].

The rationale for adding larvicides to the arsenal of malaria control tools in urban areas is manifold. First, in contrast to rural areas, vector breeding habitats are generally fewer and much easier to reach in highly densely populated areas [10]. Second, the most potent malaria vector in Africa, *An. gambiae*, has been shown to exhibit exophagic behavior in some urban areas - although the majority of bites still take place indoors [23]. If this behavior intensifies over time, and therefore more biting and resting start to occur outside of homes, the efficacy of both IRS and ITNs would be reduced. Mathematical models have provided evidence that the outdoor biting rate defines what is achievable in terms of malaria reduction with IRS and ITNs [24]. LSM is one of the few strategies that could contribute to further reduce malaria when *Anopheles* are partially exophagic [14]. Third, insecticide resistance has emerged for the primary malaria vectors in many areas of the African continent [25]–[28] and combining IRS and ITN with larviciding could become more desirable in such settings. Finally, relying solely on IRS and ITNs may be insufficient to achieve malaria elimination in much of sub-Saharan Africa [29], [30]. As such, larviciding may be part of an integrated vector management (IVM) approach [31] that could help hinder malaria transmission [18]. Such informed use of larvicides, based on local malaria ecology, is in line with WHO’s current position on IVM [31], [32].

Africa is the fastest urbanizing continent in the world and its share of urban population is expected to double between 2000 and 2030 [33]. Malaria intensity is generally much lower in urban areas and transmission is highly focal [34], [35]. A corollary of this reduced endemicity is that urban dwellers will develop lower levels of clinical immunity to the disease, which can pose public health challenges. It has been estimated that about 28% of the malaria burden in sub-Saharan Africa is attributable to urban malaria [34]. Malaria control in urban settings offers more options than for rural areas because logistical constraints are alleviated by relatively good transportation, education, communication, and health infrastructures [36].

Following this rationale, the Dar es Salaam Urban Malaria Control Program (UMCP) was launched in 2004, targeting 15 of the city’s 73 wards, covering 56 km^2^ of the city, and a population of more than 610,000 residents [36]. The goal was to develop a sustainable larval control intervention as one of the main components of a malaria control strategy. Regular application of microbial larvicides was initiated in 2006 through vertically managed community-based delivery systems [36]. Initial results, restricted to children under five years of age and comprising data from the first period of larviciding (2006–2007) in three wards of the city (N = 4,450), demonstrated that this intervention reduced by 72% the odds of malaria infection [21]. In addition, rigorous monitoring of larval population in the same period showed that larviciding reduced anopheline larval abundance by 96% [36]. The larviciding intervention was scaled-up to 9 wards in 2007 and to all 15 wards in 2008.

In this paper, we will comprehensively investigate the effectiveness of the larviciding intervention on reducing malaria prevalence using 4.6 years of data, including individuals of all ages, and combining both cross-sectional and longitudinal data (N = 64,537). This will provide crucial evidence on the potential contribution of larvicide use for reducing population-level malaria burden in urban areas of sub-Saharan Africa.

## Materials and Methods

### Study site

**Figure 1:**
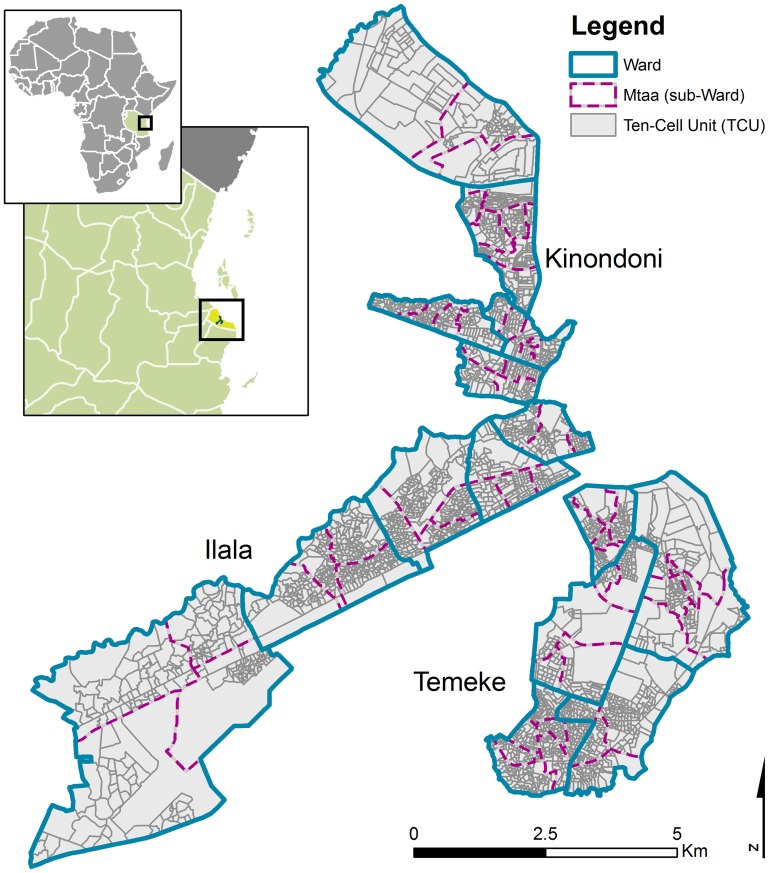
Map of the study area and administrative units. The northern portion belongs to the municipality of Kinondoni, the south-eastern portion to Temeke, and the south-western part to Ilala.

Dar es Salaam is the largest city and economic capital of the United Republic of Tanzania with an estimated population of 2.7 million in 2005 [37]. The climate is tropical humid with two rainy seasons – the long rains during the months of April and May and the short rains of October and November. Malaria transmission is year-round [38] with peaks in incidence after the two rainy seasons. *Plasmodium falciparum* accounts for more than 90% of cases and the principal vectors involved in malaria transmission are *An. gambiae s.s*. and *An. funestus* [10]. *An. coustani*’s contribution to malaria transmission is believed to be marginal [21]. Dar es Salaam is composed of three municipalities: Illala, Temeke, and Kinondoni. These municipalities are further divided in 73 wards (Figure 1). Each ward is comprised of administrative sub-units called *mtaa* (plural *mitaa*) which are further divided in ten-cell units (TCU) – the smallest administrative unit that contains approximately 10–20 houses, but may also contain as many as 100 [10].

### Design of the larviciding intervention

**Figure 2:**
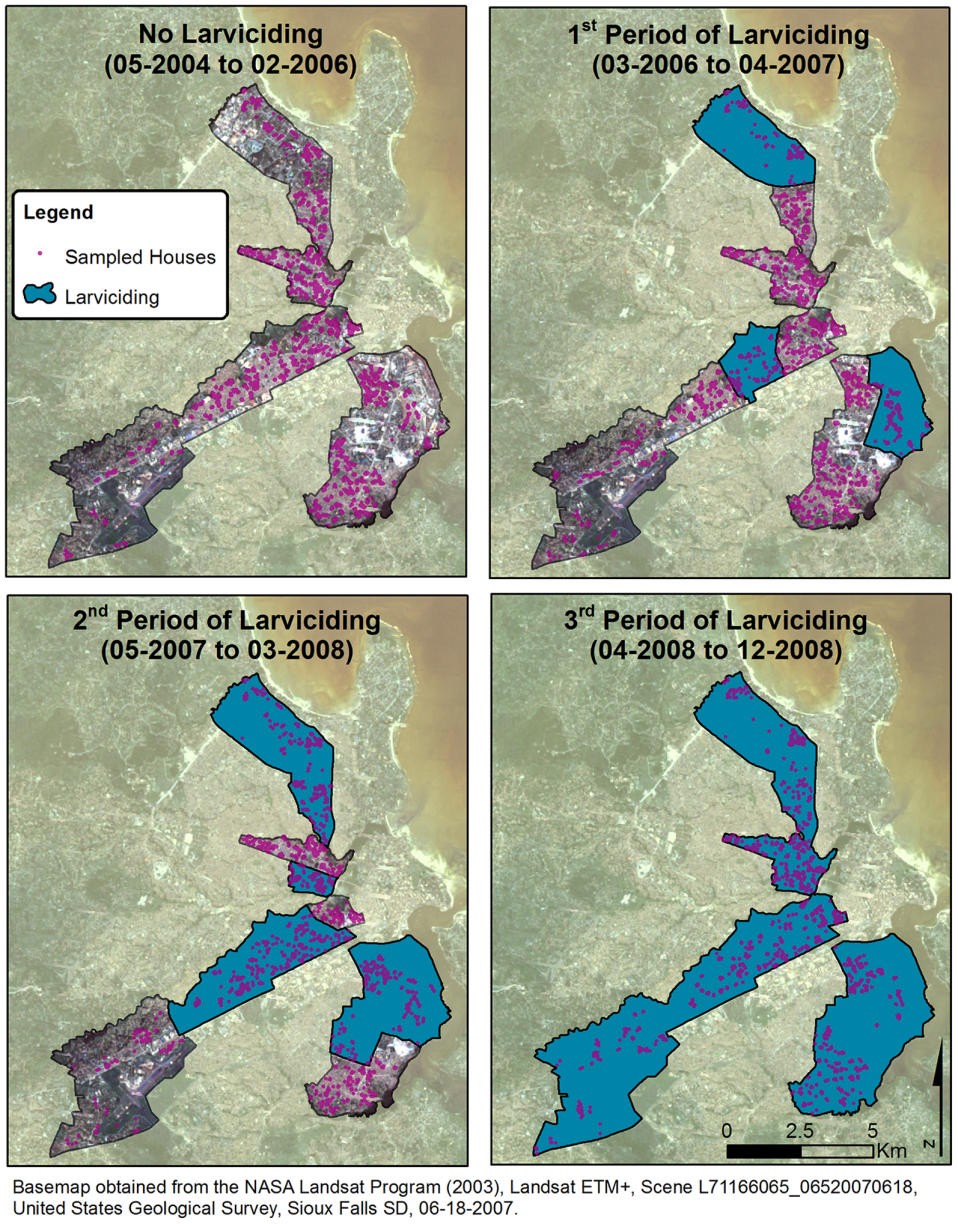
Map control and intervention wards and location of sampled households for each larviciding period.

The Dar es Salaam’s UMCP was launched in 2004, and targeted 15 wards, five in each of the three municipalities, totaling 67 *mitaa*. During the first phase of the project (May 2004 to February 2006), systems for extensive mapping [39], [40] and surveillance of potential mosquito breeding sites were developed [36]. In 2005, routine surveillance of immature and adult mosquitoes was fully operationalized. Comprehensive larviciding of the identified breeding habitats debuted in March 2006 in three wards (Figure 2). The program was community-based but the UMCP remained responsible for vertical management and supervision. This entailed that responsibility for routine mosquito control and surveillance was delegated to modestly paid community members referred to as Community-Owned Resource Person (CORP) [11], [12], [41]. After 13 months of larviciding in these three wards, operations were extended to six additional wards: two in each municipality, totaling 9 wards covered by larviciding activities. Finally, about 12 months later, in April of 2008, the intervention was scaled-up to all 15 wards of the UMCP. The order in which wards were chosen to receive the larviciding intervention was not randomly allocated. Rather, the choice was the result of careful consideration of the following two criteria: (i) the availability of comprehensive and detailed maps of the ward, and (ii) the proven ability of the ward supervisor and CORPs to efficiently undertake the required tasks.

The biological agents *Bacillus thuringiensis* var. *israelensis* (*Bti*; VectoBac® Valent BioSciences Corporation, VBC, USA) and *Bacillus sphaericus* (*Bs*; VectoLex®, VBC, USA) were used to control the aquatic stages of anopheline mosquitoes. Each *mtaa,* or portion of a *mtaa*, was under the responsibility of a designated CORP who was instructed to treat breeding habitats on a weekly basis. The dosage was 0.04 grams per m^2^ and 1 gram per m^2^ for *Bti* and *Bs,* respectively. Closed habitats that mainly breed *Culex quinquefaciatus* were treated with *Bs* every three months by a separate team of CORPs (although *Culex* mosquitoes play no role in malaria transmission, this was a programmatic decision to gain support from the community).

### UMCP Data collection

**Figure 3:**
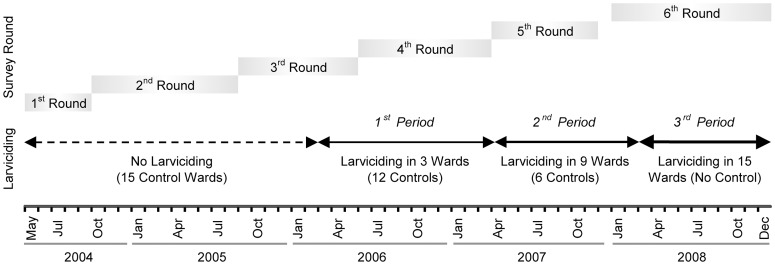
Timeline of data collection activities and larviciding intervention. The first survey round was conducted form 05/2004 to 09/2004, the second from 10/2004 to 08/2005, the third from 09/2005 to 05/2006, the fourth from 06/2006 to 03/2007, the fifth from 04/2007 to 11/2007, and the sixth and last survey round from 01/2008 to 12/2008. The first period of the intervention started on March 1^st^ 2006, the second period of larviciding on May 1^st^ 2007, and the last period of larviciding on April 1^st^ 2008.

During the study period, a total of six randomized cluster-sampled household surveys were carried out (Figure 3). A list of TCUs was assembled for each ward before March of 2004 and was regularly updated throughout the study duration. During the first round of the survey, ten TCUs were randomly sampled from each of the 15 wards. All households located in the sampled TCUs were invited to participate in the survey. From the second round onwards, the TCUs sampled in the first round were followed-up longitudinally, and another ten TCUs per ward were selected for cross-section surveys. Since loss to follow-up is non-negligible in urban areas, starting from the 3^rd^ survey round, the list of subjects to be followed-up also included randomly selected subjects interviewed in previous cross-section surveys. This was implemented in order to guarantee that the minimum required sample size would be met. Sample size calculations used a significance level of 5% and 80% power to detect a 5% absolute difference in malaria prevalence from 10% baseline prevalence. This is equivalent to a ±50% relative risk of infection. Calculations were based on mean TCU population size [21].

Upon consenting to the interview, each household was geo-referenced using a hand-held global positioning system (GPS) device. A detailed questionnaire was administered, collecting information grouped in four modules: (i) house characteristics (e.g., location, conditions, number of habitants); (ii) head of the household (e.g., occupation, education, knowledge of malaria transmission and disease symptoms, assets, agricultural practices); (iii) use of preventive measures (e.g., bednet, mosquito repellent, coil); and (iv) individual characteristics (e.g., age and sex of all household members, occurrence of fever in the past two weeks, treatment-seeking behavior, use of antimalarial drug, sleeping habits, travel history). A proxy for socio-economic status was constructed using an asset-based index calculated by performing Principal Component Analysis [42] of the households’ possession, excluding protective assets such as bednets and window screenings. Table 1 describes the variables selected for this study, their type and, if appropriate, the way they were categorized.

**Table 1:** Characteristics of study participants stratified by survey round and intervention group (lagged by 5 weeks).

Variables		Survey round #1	Survey round #2	Survey round #3		Survey round #4		Survey round #5		Survey round #6	
		Control	Control	Control	Larvicide	Control	Larvicide	Control	Larvicide	Control	Larvicide
***Outcome:*** Prevalence of malaria infection		20.8%	16.9%	10.2%	13.1%	7.1%	4.6%	5.2%	4.4%	2.3%	1.7%
***Individual-Level Variables***		**n = 5,809**	**n = 11,149**	**n = 10,791**	**n = 697**	**n = 9,951**	**n = 2,385**	**n = 6,461**	**n = 5,663**	**n = 744**	**n = 10,887**
Age											
	0 to <5 years of age	16.0%	14.9%	15.3%	15.1%	13.5%	12.7%	12.3%	11.5%	18.4%	10.3%
	5 to <15 years of age	27.7%	27.7%	27.2%	30.1%	28.3%	28.9%	28.0%	31.0%	26.7%	30.3%
	15 to <30 years of age	28.5%	29.2%	28.4%	29.1%	29.0%	29.3%	28.6%	28.6%	30.4%	29.3%
	30 to <45 years of age	15.8%	16.3%	16.8%	14.5%	17.0%	16.6%	19.2%	18.7%	14.7%	18.4%
	45 to <60 years of age	7.1%	7.2%	7.2%	8.3%	7.3%	7.6%	7.8%	6.6%	5.2%	7.5%
	≥ 60 years of age	4.9%	4.7%	5.1%	2.9%	4.8%	4.9%	4.2%	3.7%	4.6%	4.2%
	Missing	0.2%	0.2%	0.1%	0%	0.1%	0%	0%	0%	0%	0%
Place slept in previous two weeks											
Outside the ward		2.9%	2.1%	6.2%	12.1%	8.4%	9.3%	4.7%	8.5%	29.2%	5.1%
Missing		0.1%	0.1%	0.1%	0%	0.2%	0.1%	0%	0%	0%	0.1%
Male sex		36.7%	35.0%	34.5%	35.4%	35.3%	37.0%	36.2%	38.4%	35.2%	39.0%
Slept under a bed net the night before		78.7%	88.9%	85.3%	97.6%	87.8%	78.9%	86.0%	82.2%	94.2%	91.5%
Slept under an ITN the night before		20.5%	23.4%	27.8%	23.7%	24.8%	20.5%	20.9%	20.7%	14.2%	29.3%
Use of coil the night before		4.9%	5.8%	6.6%	8.9%	7.4%	5.1%	8.6%	8.0%	2.2%	5.7%
Use of repellent the night before		0.3%	1.3%	1.6%	4.9%	5.0%	3.4%	3.0%	3.0%	0.5%	3.3%
Use of spray the night before		8.4%	10.5%	15.8%	16.8%	21.0%	18.2%	30.8%	30.6%	6.6%	29.2%
Took malaria drug in previous two weeks		7.4%	3.7%	5.4%	3.0%	8.2%	3.9%	4.9%	6.9%	6.3%	2.0%
Interviewed during wet season		10.7%	49.7%	56.2%	100.0%	27.4%	37.5%	64.2%	12.8%	99.5%	46.5%
Follow-up observation		0%	17.0%	26.5%	24.1%	31.2%	32.1%	35.2%	32.8%	17.2%	27.2%
***Household-Level Covariates***		**N = 1,240**	**N = 2,107**	**N = 2,038**	**N = 124**	**N = 1,824**	**N = 396**	**N = 1,046**	**N = 827**	**N = 103**	**N = 1,549**
Occupation of household head/designated											
	Business / Government / Formal sector	63.1%	58.2%	59.8%	67.7%	67.0%	64.4%	60.7%	68.0%	37.9%	76.7%
	Farmer / Fisherman	3.3%	1.6%	2.1%	0.0%	0.9%	2.0%	1.4%	0.7%	0%	0.8%
	Informal sector	16.9%	17.8%	21.1%	22.6%	19.7%	16.7%	22.8%	17.9%	53.4%	12.5%
	Retired / No job / Domestic	15.2%	20.5%	16.3%	9.7%	11.3%	15.2%	13.3%	12.9%	7.8%	9.0%
	Missing	1.5%	1.9%	0.8%	0%	1.0%	0.8%	1.7%	0.5%	1.0%	1.0%
Socio-Economic Status											
	Lowest quintile	32.0%	32.3%	29.7%	12.9%	20.4%	24.0%	7.3%	7.3%	3.9%	8.4%
	Second quintile	29.4%	28.7%	26.2%	20.2%	23.6%	15.4%	20.9%	16.3%	11.7%	15.0%
	Third quintile	13.6%	12.1%	16.0%	20.2%	19.9%	18.9%	14.1%	15.7%	57.3%	18.1%
	Fourth quintile	12.1%	11.2%	12.5%	21.8%	19.7%	19.9%	29.2%	30.7%	23.3%	29.1%
	Highest quintile	12.9%	15.7%	15.5%	25.0%	16.3%	21.7%	28.5%	30.0%	3.9%	29.4%
Education of Household Head/Designated											
	Illiterate	6.0%	4.5%	9.4%	0.8%	6.4%	5.3%	2.6%	2.7%	13.6%	1.6%
	Primary	64.4%	60.6%	51.0%	50.0%	46.2%	48.0%	35.9%	30.8%	48.5%	35.6%
	Secondary	26.9%	28.2%	33.3%	37.9%	42.0%	39.6%	57.4%	60.2%	37.9%	59.3%
	Tertiary	1.7%	3.6%	4.9%	11.3%	4.5%	5.8%	3.4%	5.4%	0%	2.9%
	Other	0.2%	0.2%	0.4%	0%	0.1%	0.5%	0%	0%	0%	0.1%
	Missing	1.0%	2.9%	1.0%	0%	0.9%	0.8%	0.7%	0.8%	0%	0.6%
Know how malaria is transmitted		68.7%	62.4%	78.4%	83.9%	82.9%	84.3%	90.2%	90.1%	81.6%	88.6%
House has window screening		22.0%	19.7%	29.5%	37.9%	23.7%	48.0%	21.5%	28.3%	31.1%	39.1%
House has complete ceiling		27.6%	24.8%	24.1%	35.5%	29.4%	36.4%	42.4%	46.8%	14.6%	33.2%
Own house		51.9%	63.1%	72.4%	66.1%	76.4%	80.3%	81.2%	80.2%	85.4%	85.7%
Household cultivates crops		19.4%	11.0%	10.3%	12.1%	8.7%	11.4%	5.8%	6.8%	13.6%	5.6%

Malaria infection status was ascertained for all household members for whom written informed consent was provided. Finger-pricked blood samples were analyzed using Giemsa-stained thick smear microscopy. Quality check was conducted on a 10% sample of blood slides at the Muhimbili University of Health and Allied Sciences – MUHAS (a center of excellence in laboratory analysis), indicating a 94.5% specificity rate and 95.7% sensitivity rate [43]. Individuals found to be infected with malaria were treated with appropriate front-line regimens (sulphadoxine-pyrimethamine until August 2006, after which it was replaced with artesunate-amodiaquine). In order to minimize selection bias and achieve full coverage for each house and TCU, up to three attempts were made to enroll subjects.

Information was collected from a total of 48,525 unique individuals and the great majority of them (39,146) were interviewed once. A total of 5,223 participants were followed up twice, 2,349 three times, 1,236 four times, 472 five times, and 99 subjects participated in every round of the survey. Including follow-up data, our sample is thus composed of 64,537 observations, which were drawn from 913 unique TCU and 6,796 households. The small number of subjects who participated in more than two rounds results from two main factors. First, the high mobility observed among urban dwellers; in the second survey round 25.6% of the subjects had moved or were travelling. Second, 13.9% of those interviewed in round 1 declined to participate in the second survey round. Reasons for refusal included pain inflicted by the finger prick, misconceptions about malaria transmission, and the mistrust of the malaria counts provided in the precedent survey round. Sensitization efforts addressed these issues and refusal decreased in subsequent survey rounds.

### Rainfall data

Rainfall estimates were obtained from the National Oceanic and Atmospheric climate prediction center. This data source combines modeling of satellite-based infrared data collected each 30-minute and station rainfall data to estimate the quantity of daily precipitation over the African continent, and has a spatial resolution of 8 kilometers [44]. Given the biology of the *Anopheles* mosquito and of the *Plasmodium* parasite, the effect of rainfall on malaria transmission is expected to be lagged in time. Previous empirical studies suggested that the effect of rainfall on malaria transmission is lagged by approximately 8 weeks [45]–[47]. For each observation, we therefore calculated total weekly precipitation (cm) and lagged this estimate by 8 weeks.

### Statistical analyses

The main outcome for this study is malaria infection status (a binary variable – Table 1) as determined by the Giemsa-stained thick smear. Malaria transmission is most directly related to the density of sporozoites-infected adult anophelines, which are not targeted by the larviciding activities. Therefore, a decline in the prevalence of malaria infection is not expected to be observed until the existing pool of infected mosquitoes dies off, and the overall density of mosquitoes is reduced. Based on observations of entomological indices and malaria incidence, it has been estimated that peaks in vector density are followed by peaks in malaria incidence after approximately 1–2 months [48]. Also, the implementation of larviciding activities requires fine-tuning before CORPs became fully familiar with the routine procedures, which could further lag any potential impacts. Based on programmatic and biological considerations, a lag of five weeks was deemed most appropriate and is consistent with results from a previous larviciding study in urban Cameroon [49].

The effects of the microbial larviciding activities on malaria occurrence were first examined using univariate statistics. Malaria prevalence was calculated for each survey round, stratifying by larviciding intervention status, if applicable. Confidence intervals for malaria prevalence were constructed using 9,999 bootstrapped replicates. Clustering of standard errors was taken into account by defining the sampling unit as the TCU [50].

Bayesian random effects logistic models where used to take into account clustering of observations at the household and TCU levels in multivariable analyses. We assumed that our binary outcome followed a Bernoulli distribution, *Y_i_* ∼ *Bernoulli(p_i_)*, where *p_i_* is the probability of an individual harboring malaria parasites, which is itself a function of covariates modeled with a *logit* link. Our model has the following form:
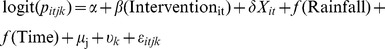





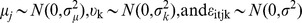



where *p_itjk_* is the probability of individual *i* at time *t* living in TCU *j* and household *k* to be infected with malaria; *β* is the coefficient of the larviciding intervention; *δ* is a vector of coefficients for control variables in vector *X* (age, sex, sleeping outside of ward in previous weeks, taking antimalarial drug in previous two weeks, individuals treated for malaria in a previous survey round, sleeping under an ITN the night before, living in a house with a complete ceiling, and living in a house with window screens) – in the case of longitudinal observations, many of these variables are time variant; *μ_j_* is a TCU-level random effect; *υ_k_* is an household random effect; and *ε_itjk_* are the residuals. Rainfall was modeled using a smooth function where the spline penalty follows a second-order random walk process (where second-order increments are assumed to be independent with mean of zero and variance *σ_t_^2^*). This is appropriate when one wants to model smooth curves with small curvatures [51], [52], which is likely to be the case for the relationship between malaria and rainfall. Finally, the time trend was accounted for with *f(.)* and modeled as a first order autoregressive process [53]. It was chosen over other type of process based on the Deviance Information Criterion (DIC) [54], which provides information on the model’s fit while penalizing for model complexity.

Potential effect modification of the intervention by other determinants of malaria infection was also investigated for a number of covariates (e.g., age, use of ITN, house proofing, etc.). Variable selection for the final multivariable models was achieved through the consideration of a number of issues: (i) subject-matter knowledge about confounders, (ii) variable exhibiting sufficient variation, and (iii) extent of potential measurement errors.

In order to investigate the robustness of our results to modeling assumptions, we used three additional model specifications by including: (i) individual random effects, (ii) ward fixed effects, and (iii) spatially-structured random effects. We also performed a number of sensitivity analyses. Specifically, we tested for potential spillover effects of the intervention, used different lags for the larviciding intervention and for the rainfall estimates, further covariate adjustments (socio-economic status, educational level, and occupation), and varied the choice of penalty for the semi-parametric time trend (first and second-order random walk). Technical details and results are presented in the Supplemental online material (Text S1).

Models were fitted using Integrated Nested Laplace Approximations (INLA) [55]. A major advantage of INLA is that it calculates posterior marginal distributions in very short computational time as compared to more traditional Markov Chain Monte Carlo (MCMC) approaches. Further, INLA has been shown to yield very high accuracy that is comparable to MCMC [55], [56]. Non-informative priors for the regression parameters and hyperparameters were used (see Text S1 for details). All analyses were performed using the R statistical software [57] and estimation of the marginal posterior distribution of the parameters of interest was performed using the *INLA* library [58]. Observations with missing data for age (n = 44), place slept in previous two weeks (n = 52), occupation of the household head (n = 134), and education level of the household head (n = 136) were retained in the analysis using the missing indicator method [59].

### Ethical considerations

Ethics approval was obtained from the Medical Research Coordination Committee of the National Institute for Medical Research, Ministry of Tanzania (Reference number NIMR/HQ/R.8a/Vol. IX/279 &234). Approval from Harvard School of Public Health Institutional Review Board was also obtained (Protocol # 20323-101). Written informed consent was obtained from all study participants after being provided with information regarding the goal, objectives, risk and benefits of the study. Parents or designated guardians provided signed informed consent on behalf of children under 18 years of age. These procedures were approved by the ethics committees.

## Results

**Figure 4:**
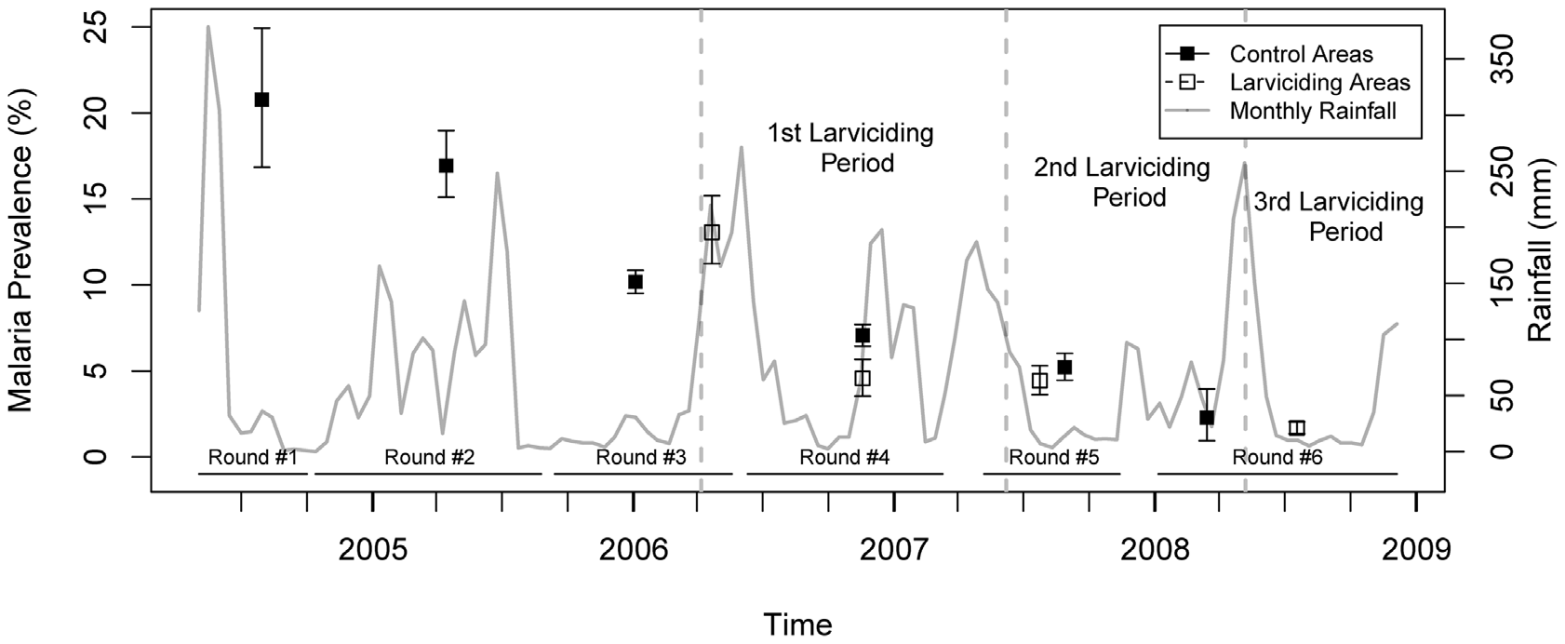
Crude prevalence of malaria infection stratified by survey round and larviciding status. Confidence intervals are based on 9,999 bootstrap replicates and account for clustering at the ten-cell unit level. Monthly rainfall variation is also shown.

Throughout the study period, malaria prevalence exhibited a considerable decline. Malaria prevalence was highest during the first round of data collection in 2004, with 20.8% prevalence (95% CI: 16.8–24.9%). It decreased to 16.9% (95% CI: 15.1–18.8%) in the second survey round, 10.4% (95% CI: 9.7–11.0%) in the third, 6.6% (95% CI: 6.0–7.1%) in the fourth, 4.8% (95% CI: 4.3–5.4%) in the fifth, and 1.7% (95% CI: 1.4–2.1%) in the last survey round. Stratifying malaria prevalence by survey round and larviciding intervention status, we observed that prevalence was slightly lower in the intervention wards as compared to the control ones, with the notable exception of the third survey round (Figure 4). Note that the start of the larviciding phases did not precisely coincide with the beginning of the survey rounds due to operational issues (as shown in Figure 3, phase 1 of larviciding was launched in March 2006, while the fourth survey round started in June 2006; phase 2 in May 2007; and phase 3 in April 2008). Hence, median dates of interviews in larviciding and control areas do not necessarily coincide, and seasonality in malaria transmission could confound the observed differences in prevalence shown in Figure 4.

For each survey round, the socio-demographic characteristics of study participants and households, stratified by larviciding intervention status, are presented in Table 1. Use of bednet was highly variable through time and seems to be correlated with rainfall and, probably, abundance of nuisance insects. The proportion of interviews performed during the wet seasons also differs between larviciding and control groups. Interestingly, the proportion of individuals reporting having taken anti-malarial drug in the previous two weeks remained relatively constant through time despite the overall decline in malaria prevalence. Finally, we note that socio-economic status seems to be increasing with time, as exhibited by the rising proportion of individuals in the upper quintiles. Overall, individuals in control and larviciding areas do not seem to differ dramatically in their socio-demographic characteristics. Most differences are observed in either the third or sixth survey rounds where the sample sizes in the larviciding and control groups, respectively, are notably smaller.

Taking into account the previously stated limitations of our univariate analysis, we present in Table 2 the results from the random effects logistic regression models that account for clustering of observations within household and TCU. These analyses suggest a significant protective effect of larviciding, with a point estimate for the odds ratio of 0.79 (95% Credible Intervals (CrI): 0.66–0.93) in both univariate and multivariable analyses. When considering potential effect modification of the larviciding intervention by season, we see that larviciding activities achieved maximum programmatic impact during the dry season (Table 3) with an odds ratio of 0.60 (95% CrI: 0.47–0.75). The dry season is defined as the months of January, February, and June through September. The effect of the larviciding intervention also had synergistic effects with other malaria protective measures such as houses with window screens (OR = 0.68; 95% CrI: 0.54–0.85), houses with complete ceiling (OR = 0.66; 95% CrI: 0.53–0.83), and using an ITN the night before (OR = 0.63; 95% CrI: 0.48–0.82). Finally, the effect of the intervention was also heterogeneous among age groups with the larviciding intervention exhibiting a greater protective effect for children under five (OR = 0.61; 95% CrI: 0.46–0.80).

**Table 2:** Univariate and multivariable effect size estimates of the larviciding intervention on malaria prevalence in Dar es Salaam, 2004–2008 (N = 64,537).

	Univariate		Multivariable	
	OR*	95% CrI†	OR*	95% CrI†
Larviciding intervention	**0.79**	**0.66**–**0.93**	**0.79**	**0.66**–**0.93**
*Age*				
Under five years of age	-	-	1.00	-
≥5 and <15 years of age	-	-	**0.82**	**(0.76**–**0.90)**
≥15 and <30 years of age	-	-	**0.67**	**(0.61**–**0.73)**
≥30 and <45 years of age	-	-	**0.60**	**(0.54**–**0.66)**
≥45 and <60 years of age	-	-	**0.55**	**(0.48**–**0.63)**
≥60 years of age	-	-	**0.47**	**(0.40**–**0.56)**
Male sex	-	-	**1.08**	**(1.01**–**1.15)**
Slept outside ward (previous 2 weeks)	-	-	0.90	(0.77–1.04)
Treated for malaria (previous round)	-	-	**0.65**	**(0.56**–**0.75)**
Took malaria drug (previous 2 weeks)	-	-	1.02	(0.90–1.16)
ITN used the night before	-	-	**0.93**	**(0.86**–**0.99)**
House has closed ceiling	-	-	0.93	(0.85–1.01)
House has window screens	-	-	**0.90**	**(0.83**–**0.98)**
*Trend for time (AR1* § *)*	Yes		Yes	
*Semi-parametric smooth for rainfall*	Yes		Yes	
*Random effects (TCU & Household)*	Yes		Yes	

Statistically significant results are bolded.

* OR  =  Odds Ratio.

† CrI  =  Credible Intervals.

§ AR1  =  First Order Autoregressive Process.

**Table 3:** Effect modification of the larviciding intervention by selected determinants of malaria prevalence in Dar es Salaam, 2004–2008 (N = 64,537).

Effect modification of the larviciding intervention by selected determinants of malaria infection (Odds Ratio and 95% Credible Intervals)*			
	Control	Larviciding	Effect of Larviciding Within Strata
Wet Season	1.00	1.06 (0.84–1.33)	1.06 (0.84–1.33)
Dry Season §	0.97 (0.69–1.10)	**0.57 (0.41**–**0.77)**	**0.60 (0.47**–**0.75)**
	Control	Larviciding	Effect of Larviciding Within Strata
No Screen	1.00	0.84 (0.70–1.02)	0.84 (0.70–1.02)
Window Screens	0.93 (0.85–1.02)	**0.80 (0.65**–**0.99)**	**0.68 (0.54**–**0.85)**
	Control	Larviciding	Effect of Larviciding Within Strata
Open Ceiling	1.00	0.84 (0.70–1.01)	0.84 (0.70–1.01)
Complete Ceiling	0.97 (0.88–1.06)	**0.78 (0.63**–**0.97)**	**0.66 (0.53**–**0.83)**
	Control	Larviciding	Effect of Larviciding Within Strata
No ITN	1.00	**0.83 (0.69**–**0.99)**	**0.83 (0.69**–**0.99)**
ITN used	0.96 (0.88–1.04)	**0.77 (0.61**–**0.96)**	**0.63 (0.48**–**0.82)**
	Control	Larviciding	Effect of Larviciding Within Strata
Aged ≥5 years	1.00	**0.83 (0.69**–**0.99)**	**0.83 (0.69**–**0.99)**
<5 years of age	1.35 (1.23–1.47)	**0.73 (0.56**–**0.94)**	**0.61 (0.46**–**0.80)**

Statistically significant results are bolded.

All models are adjusted for age, sex, sleeping outside of the ward (previous 2 weeks), being treated for malaria in a previous round, use of malaria drugs (previous 2 weeks), use of ITN, complete ceiling, window screen, precipitation, time trend. Random effects at household and TCU levels are also included.

§ Dry season is defined as the months of January, February, and June through September.

Model specifications seem to have little bearing on the estimates of the posterior marginal for the larviciding intervention (see Tables S1 and S2 in Text S1). Importantly, including fixed effects at the ward level, which would control for any time-invariant measured or unmeasured confounders of the larviciding-malaria relationship, had little impact on the point estimate of the larviciding intervention (adjusted OR = 0.80; 95% CrI:0.66–0.97).

Finally, our sensitivity analyses (see Table S3 and S4 in Text S1) demonstrated that spillover effects were not biasing our effect size estimate towards the null. As expected, effect size estimates were somewhat sensitive to variation in the assumed lag length between initiation of larviciding activities and malaria transmission but the effect remained statistically significant over lag lengths varying between 28 and 60 days. Results were also robust to changes in other model parameters.

## Discussion

This study has shown that a community-based larviciding program, centrally managed by the UMCP, provided significant protection to individuals living in areas covered by the larviciding operations. The strength of association was robust to model specifications and consistently approximated a 21% reduction in the odds of malaria infection. Further, the larviciding intervention achieved maximum effectiveness during the dry season and had synergistic effects with other protective measures such as use of ITN, houses with windows screens, and houses with complete ceilings. In addition, we found no evidence of spillover effects between intervention and control areas.

Our estimated effect size for the larviciding intervention is much lower, but not statistically different, than the one previously reported for the first larviciding period of the UMCP, where the odds ratio of living in areas treated with larvicides and being infected with malaria was estimated to be 0.28 (95% CI: 0.10–0.80) as compared to individuals living in control areas [21]. This can be explained in part by the fact that our study considered all age ranges, while Geissbühler et al [21] restricted their analysis to children under five years of age. While there is no reason to believe that larviciding should be more protective for children than for adults, since the intervention acts at the population level by reducing vector density, children might be more likely to spend evenings and nights at or close to their home, a period of the day when most of malaria transmission occurs. There is thus less potential misclassification of exposure for this age group as compared to adults, who might visit friends or spend time during evenings near high exposure areas not covered by larviciding activities. Indeed, we found that the product term between the larviciding intervention and age was statistically significant. The estimated odds ratio for the larviciding intervention was of 0.61 (95% CrI: 0.46–0.80) for children under five years of age which is closer to the one reported by Geissbühler et al [21] but insufficient to explain this differential. Another reason which could explain this difference in impact is that our analysis covered all three phases of the intervention with a total of 33 months of larviciding activities, while Geissbühler et al [21] analyzed only the first phase, when the intervention was operational in only three wards for 12 months. Analyses over a longer period may be impacted by programmatic fatigue, coupled with the potential impact that other unmeasured and/or unknown interventions could have on the prevalence of malaria infection and overall transmission dynamics (e.g., artemisin-based combination therapy – ACT started to be the first line of treatment in 2007).

Larviciding during the dry season was shown to be more effective at lowering the prevalence of malaria infection than during the rainy season (when the stratified effect was not significant). This result is especially interesting since 49% of malaria cases were sampled during the dry season. Since larval habitats are less numerous and easier to access when rainfall is low, larviciding activities could have been more effective at suppressing larval production due to operational issues. This highlights one of the key aspects of successful larviciding programs: the ability to locate and access all potential breeding habitats in the targeted area. Also, larviciding should not be deployed alone, but in conjunction with other appropriate vector control activities [60]. The fact that we have estimated larviciding to be more effective than ITNs in Dar es Salaam should not be taken at face value, since the effect size estimate for ITNs does not take into account potential community effects that extend to non-users [61], [62], and that the use of ITNs and other protective measures is likely a function of perceived risk by household members. The combination of different vector control strategies is also supported by our findings of significant synergistic effects between larviciding and use of ITNs, window screens, and houses with a complete ceiling.

With renewed impetus for the long-term goal of malaria eradication [63], the need for tailored programs is imperative, including vector control [30]. Vector control programs should not be established as stand-alone entities. Rather, intersectoral collaboration, health system strengthening, and community mobilization are instrumental to vector control program success. Integrated Vector Management (IVM), as endorsed by WHO [31], [64], emphasizes rational decision making processes to efficiently use resources and attain health-based targets [65]. IVM specifically acknowledges that a ‘one size fits all’ strategy for malaria control will be ineffective. Larviciding should be considered as part of an IVM approach in other urban areas of sub-Saharan Africa, if the local malaria ecology warrants its use. Our study provides a number of important lessons regarding the implementation of larval control: (i) breeding habitats can, and should, be mapped at high resolution using low-cost technology [36], (ii) locally relevant entomological information should be collected to inform operational activities, (iii) monitoring and evaluation systems should be implemented to ensure effective and appropriate delivery and fine-tuning of interventions, and (iv) community involvement and sensitization can be beneficial to programmatic activities. Other strategies included in an IVM approach could facilitate the use of larviciding. For example, in Dar es Salaam 33% of *Anopheles* breeding habitats are found in clogged drains [66]. In this context, the use of environmental management to restore the functionality of drains would result in fewer breeding habitats [43], and therefore reduce the area to be covered with larviciding.

Strengths of this study include its large sample size, longitudinal design, large temporal and spatial extent of larviciding activities that limited potential spillover effects, and availability of reliable baseline information. This study also has some limitations. First, the wards targeted by the UMCP were not randomly allocated to the larviciding intervention. This entails that our effect size estimates for the larviciding intervention could be biased by residual confounding. This is unlikely to be the case as including fixed effects at the ward level, which would control for such time-invariant non-measured confounders, did not impact our results. Second, ACTs were effectively introduced in Dar es Salaam in January 2007. With its gametocidal proprieties, this drug, if used on a large scale, has the potential to significantly reduce the reservoir of malaria in the general population. Although attempts were made at collecting information on ACT use from health facility data, we were not able to assemble reliable temporal information for the targeted 15 wards. Thus, some of the secular decline in the prevalence of malaria infection observed in control areas before the introduction of larviciding may be a result of ACT use (and possibly of other unobserved activities that could potentially impact the risk of malaria transmission).

Our results have important implications for malaria control in sub-Saharan Africa. Specifically, we have provided evidence that a community-based application of microbial larvicides was effective in reducing malaria transmission in urban Dar es Salaam. Microbial larvicides have been shown to be environmentally safe, specific in their action, and highly effective in killing *Anopheles* larvae under field conditions [67]–[69]. With important projected increases in urban population in sub-Saharan Africa, mosquitoes’ behavioral adaptation to current control strategies, and the already recorded emergence of resistance to pyrethroid insecticides, larval source management, and larviciding in particular, should be given careful consideration by managers of malaria control programs.

## Supporting Information

Text S1 **Supplemental online information.** Description of results from additional model specifications (Tables S1 and S2), potential spillover effects (Table S3), sensitivity analyses performed (Table S4), and detailed information on the prior distributions used.
(DOC)
