# Supplementary material for: Impact of Community-Based Larviciding on the Prevalence of Malaria Infection in Dar es Salaam, Tanzania
Source: PLoS One. 2013 Aug 14;8(8):e71638. doi: 10.1371/journal.pone.0071638 (PMC3743749; doi:10.1371/journal.pone.0071638)
Supplement: Text S1 — Supplemental online information. Description of results from additional model specifications (Tables S1 and S2), potential spillover effects (Table S3), sensitivity analyses performed (Table S4), and detailed information on the prior distributions used. (DOC) [file pone.0071638.s001.doc]

**Supplemental online material:**

Impact of Community-Based Larviciding on the Prevalence of Malaria Infection in Dar es Salaam, Tanzania

*Mathieu Maheu-Giroux & Marcia C. Castro*

In order to assess the robustness of our results three additional model specifications were used and a number of sensitivity analyses were performed. This supplemental material is organized in four sections. First we describe three additional model specifications used for obtaining effect size estimates for the larviciding intervention. Second we present the parameters for which we performed the sensitivity analyses. Third, the different prior distributions for the models’ parameters and hyperparameters are defined. Lastly, results from the different models and analyses are briefly discussed.

**Methods**

*Allowing for different model specifications*

As some individuals were followed-up longitudinally in time, we first included a random effect at the individual level as an additional model specification. Further, because the order of the roll-out of the larviciding intervention was not randomized, we cannot eliminate the possibility that ward characteristics are correlated with the intervention. We thus used a second model specification that includes ward fixed effects. Finally, observations may also be spatially dependent given the focal nature of urban malaria. Spatially-structured random effects were hence included in the third model specification to allow for such spatial autocorrelation. Again, we assumed that the binary outcome followed a Bernoulli distribution, *Y_i_* ~ *Bernoulli(p_i_)*, where *p_i_* is the probability of an individual harboring malaria parasites, which is itself a function of covariates modeled with a *logit* link. The three additional models have the following form:

Model (1): TCU, household, and individual levels random effects model

$\text{logit}(p_{itjk})=\text{ }\alpha+\beta(\text{Intervention}_{\text{it}})+\delta\text{ }X_{it}+f(\text{Rainfall)}+f(\text{Time})+\mu_{\text{j}}+\upsilon_{k}+m_{i}+\varepsilon_{itjk}$ $\mu_{j}\sim N(0,\sigma_{\mu}^{2}),\text{ }\upsilon_{\text{k}}\sim N(0,\sigma_{k}^{2})\text{, }m_{i}\text{\textasciitilde}N(0,\sigma_{i}^{2}\text{), and }\varepsilon_{\text{itjk}}\sim N(0,\sigma^{2})$,

where *p_itjk_* is the probability of individual *i* at time *t* living in TCU *j* and household *k* to be infected with malaria; *m_i_* is an individual-level random effect; *β* is the coefficient of the larviciding intervention; *δ* is a vector of coefficients for control variables in vector *X*; *μ_j_* is a TCU-level random effect; *υ_k_* is an household random effect; and *ε_itjk_* are the residuals. Rainfall was modeled using a smooth function where the spline penalty follows a second-order random walk process (where second-order increments are assumed to be independent with mean of zero and variance *σ_t_^2^*). Finally, the time trend was accounted for with *f(.)* and modeled as a first order autoregressive process [1]. Individual-level random effects were included to account for subjects followed-up in two or more surveys.

Model (2): Household and TCU random effects with ward fixed effects

$\text{logit}(p_{itjk})=\text{ }\alpha+\beta(\text{Intervention}_{\text{it}})+\delta\text{ }X_{it}+f(\text{Rainfall)}+f(\text{Time})+\omega\text{Ward}_{it}+\mu_{\text{j}}+\upsilon_{k}+\varepsilon_{itjk}$ $\mu_{j}\sim N(0,\sigma_{\mu}^{2}),\text{ }\upsilon_{\text{k}}\sim N(0,\sigma_{k}^{2})\text{, and }\varepsilon_{\text{itjk}}\sim N(0,\sigma^{2})$,

where *p_itjk_* is the probability of individual *i* at time *t* living in TCU *j* and household *k* to be infected with malaria; ω is a vector of coefficients for the ward fixed effects; and *β, δ*, *f(.)*, *μ_j_*, *υ_k_*, and *ε_itjk_* are similar to those described in Model (1).

Model (3): Household-level and spatial random effects model

For the spatial random effects model, the high number of observations and computing limitations made model fitting problematic. To reduce the dimensionality of the data, observations were grouped at the household level and a binomial distribution was assumed, *Y_k_* ~ *Binomial(p_k_, n_k_,)*, where *Y_k_* is the number of malaria cases in household *k*, *n_k_* is the number of individuals in household *k,* and *p_k_* is the probability being infected with malaria for individuals living in household *k*. Specifying the model using this binomial logistic regression framework greatly reduced computing time. The trade-off was that we are no longer able to control for individual-level covariates although the effect size estimate for the larviciding intervention can still be interpreted at the individual level. The model was defined as:

$$\text{logit}(p_{kt})=\text{ }\alpha+\beta(\text{Intervention}_{\text{kt}})+\delta\text{ }X_{it}+f(\text{Rainfall)}+f(\text{Time})+\upsilon_{k}+\rho_{k}+\varepsilon_{tk}$$

$\text{ }\upsilon_{\text{k}}\sim N(0,\text{ }\sigma_{k}^{2})\text{, }\rho_{\text{k}}\left|\rho_{k\neq s}\right.\sim N(\frac{\sum_{k\neq s} w_{ks}\rho_{k}}{\sum_{k\neq s} w_{ks}},\frac{\sigma_{r}^{2}}{\sum_{k\neq s} w_{ks}}\text{), and }\varepsilon_{\text{tk}}\sim N(0,\text{ }\sigma^{2})$,

where *p_kt_* is the probability of being infected with malaria for individual living in household *k* at time *t;* *υ_k_* is a household random effect; *w_ks_* is a binary spatial weight matrix; and ρ*_k_* is a spatial random effect that follows a Gaussian Conditional Autoregressive (CAR) distribution, as proposed by Besag [2]. CAR models have the advantage of providing good approximations of continuous geostatistical processes and are more statistically efficient than geostatistical models [3,4]. CAR models belong to the family of spatial error models, which aim at uncovering causal relationships and assume that spatial dependence occurs because of omitted and spatially correlated variables [1]. In the present case, such omitted variables could be related to mosquito dispersion and behavior, unmeasured human behaviors, unreported programmatic challenges, undocumented local environmental characteristics/changes, or other isolated efforts to control malaria unknown to the UMCP. The neighborhood for the spatial weight matrix is defined based on the distance beyond which residuals are not spatially correlated. Based on visual inspection of Model’s (1) residuals and considerations of anophelines dispersion in urban environments, a distance threshold of 200 meters was deemed most appropriate. Indeed, previous studies have shown that malaria transmission in urban environment is highly focal and that adult anophelines do not disperse more than 200-300 meters from larval habitats in urban settings [5-8]. Further, a study conducted in urban Ouagadougou (Burkina Faso) indicated that *P. falciparum* infections were clustered within 200 meters of larval habitats [9].

*Sensitivity analyses*

To assess if potential spillover effects were biasing our effect size estimates, two binary variables were created: (i) being in a control ward and within 100 meters of an intervention ward (spillover from intervention to control wards), and (ii) being in an intervention ward and within 100 meters of a control ward (spillover from control to intervention areas). Given the focal transmission of malaria in urban settings and the overall distribution of TCU sizes, a distance threshold of 100 meters in both directions was felt most appropriate. These binary variables were included as covariates along with the larviciding intervention in the regression model.

Further sensitivity analyses were performed by investigating the effect of using different lags for the intervention (28 days, 35 days, 45 days, and 60 days), the influence of the model’s choice to account for the time trend (first-order autoregressive, first-order random walk, and second-order random walk), and the impact of different lags for rainfall (7 weeks, 8 weeks, and 9 weeks) on the effect size estimate for the larviciding intervention. Additionally, we present the effect size estimates for the larviciding intervention using further adjustments with the following covariates: quintiles of socio-economic status, occupation of the household head, and education level of the household head.

*Prior specifications*

All priors for the regression parameters were assumed to have non-informative Gaussian distributions with mean of zero and precision of 0.001 (precision=1/variance). Priors for the precision hyperparameters of the random effects at the individual, household and TCU level and spatially structured random effect were defined on the logarithmic scale and assumed to follow non-informative logGamma (shape = 0.001, scale = 0.001) distributions. The log-precision of the rainfall and time semi-parametric smooth were given logGamma(shape = 1, scale = 1e-5) priors as proposed by Natário and Knorr-Held [10]. The first lag correlation parameter of the first order autoregressive process is defined on the logit scale and was given a Gaussian (mean = 0, precision = 0.4) prior for the first lag correlation parameter (which was defined on the logit scale).

Estimation of the marginal posterior distribution of the parameters of interest was performed using *Integrated Nested Laplace Approximations* [11] and the *INLA* library [12] in R was used for model fitting. Observations with missing data for age (n=44), place slept in previous two weeks (n=52), occupation of the household head (n=134), and education level of the household head (n=136) were retained in the analysis using the missing indicator method [13].

**Results and Discussion**

Model’s specifications seem to have little bearing on the estimates of the posterior marginal for the larviciding intervention (Table S1). Including a random effect at the individual level had no effect on marginal posterior for the larviciding intervention (Odds Ratio (OR)=0.78; 95% Credible Interval (CrI): 0.66-0.93). Importantly, including fixed effects at the ward level, which would control for any time-invariant measured or unmeasured confounders of the larviciding-malaria relationship, had little impact on the point estimate of the larviciding intervention (OR=0.81; 95% CrI: 0.67-0.97). The credible interval was somewhat larger, however. This can be explained by the fact that fixed effects disregard the between wards variation in exposure and is thus less statistically efficient than the other models. Finally, allowing for spatially structured random effects did not impact the effect size estimate for the larviciding intervention.

**Table S1:** Unadjusted effect size estimates of the larviciding intervention on malaria prevalence in Dar es Salaam, 2004-2008 (N=64,537).

|  | **Model (1)** | | **Model (2)** | | **Model (3)** | |
| --- | --- | --- | --- | --- | --- | --- |
|  | **OR*** | **95% CrI†** | **OR*** | **95% CrI†** | **OR*** | **95% CrI†** |
| Larviciding intervention | **0.78** | **(0.66-0.93)** | **0.81** | **(0.67-0.97)** | **0.78** | **(0.66-0.91)** |
|  |  |  |  |  |  |  |
| *Trend for time (AR1*§*)* | Yes | | Yes | | Yes | |
| *Random effects* |  | |  | |  | |
| TCU & household | Yes | | Yes | | - | |
| Individual | Yes | | - | | - | |
| Household & spatial | - | | - | | Yes | |
| *Fixed effects at ward level* | - | | Yes | | - | |
|  |  | |  | |  | |

Statistically significant results are bolded

*OR = Odds Ratio

†CrI = Credible Intervals

§AR1 = First Order Autoregressive Process

Adjusting these three models for potential confounders of the larviciding-malaria relationship has shown that our effect size estimate was robust to such adjustments (Table S2). Odds ratio for the larviciding intervention ranged from 0.79 (95% CrI: 0.66-0.94) to 0.80 (95% CrI: 0.66-0.97).

**Table S2:** Adjusted effect size estimates of the larviciding intervention on malaria prevalence in Dar es Salaam, 2004-2008 (N=64,537).

|  | **Model (1)** | | | **Model (2)** | | | **Model (3)** | |
| --- | --- | --- | --- | --- | --- | --- | --- | --- |
|  | **OR*** | **95% CrI†** | **OR*** | | **95% CrI†** | **OR*** | | **95% CrI†** |
| Larviciding intervention | **0.79** | **(0.66-0.94)** | **0.80** | | **(0.66-0.97)** | **0.79** | | **(0.67-0.93)** |
| *Age* |  |  |  | |  |  | |  |
| Under five years of age | 1.00 | - | 1.00 | | - | - | | - |
| ≥5 and <15 years of age | **0.82** | **(0.76-0.90)** | **0.83** | | **(0.76-0.90)** | - | | - |
| ≥15 and <30 years of age | **0.67** | **(0.61-0.73)** | **0.67** | | **(0.62-0.74)** | - | | - |
| ≥30 and <45 years of age | **0.60** | **(0.54-0.66)** | **0.60** | | **(0.54-0.67)** | - | | - |
| ≥45 and <60 years of age | **0.55** | **(0.48-0.63)** | **0.55** | | **(0.48-0.63)** | - | | - |
| ≥60 years of age | **0.47** | **(0.39-0.56)** | **0.47** | | **(0.40-0.56)** | - | | - |
| Male sex | **1.08** | **(1.01-1.15)** | **1.08** | | **(1.02-1.15)** | - | | - |
| Slept outside ward (previous 2 weeks) | 0.90 | (0.77-1.04) | 0.88 | | (0.76-1.02) | - | | - |
| Treated for malaria (previous round) | **0.65** | **(0.56-0.75)** | **0.65** | | **(0.56-0.75)** | - | | - |
| Took malaria drug (previous 2 weeks) | 1.02 | (0.89-1.16) | 1.01 | | (0.89-1.15) | - | | - |
| ITN used the night before | **0.93** | **(0.86-0.99)** | **0.92** | | **(0.86-0.99)** | - | | - |
| House has closed ceiling | 0.93 | (0.85-1.01) | 0.94 | | (0.87-1.03) | **0.89** | | **(0.81-0.96)** |
| House has window screens | **0.90** | **(0.82-0.98)** | **0.90** | | **(0.82-0.98)** | **0.89** | | **(0.81-0.97)** |
|  |  |  |  | |  |  | |  |
| *Trend for time (AR1*§*)* | Yes | | | Yes | | | Yes | |
| *Semi-parametric smooth for rainfall* | Yes | | | Yes | | | Yes | |
| *Random effects* |  | | |  | | |  | |
| TCU & household | Yes | | | Yes | | | - | |
| Individual | Yes | | | - | | | - | |
| Household & spatial | - | | | - | | | Yes | |
| *Fixed effects at ward level* | - | | | Yes | | | - | |
|  |  | | |  | | |  | |

Statistically significant results are bolded

*OR = Odds Ratio

†CrI = Credible Intervals

§AR1 = First Order Autoregressive Process

Potential spillover effects from either control to larviciding areas or larviciding to control areas would bias our effect size estimates towards the null. After including indicator variables for being within a 100 meters buffer zone in both directions (i.e., control to intervention and intervention to control), we found no evidence of spillover effects as the odds ratio for the larviciding intervention remained virtually unchanged (Table S3).

**Table S3: Investigation of potential spillover effects from larviciding to control areas and from control to larviciding areas in Dar es Salaam, 2004-2008 (N=64,537).**

| **Variables**§ | **OR*** | **95% CrI†** |
| --- | --- | --- |
| Larviciding intervention | **0.77** | **(0.65-0.92)** |
| Spillover from control to larviciding areas (100 m buffer) | 1.10 | (0.82-1.47) |
| Spillover from larviciding to control areas (100 m buffer) | 0.71 | (0.33-1.44) |
|  |  |  |

Statistically significant results are bolded

*OR = Odds Ratio

†CrI = Credible Intervals

§ All models are adjusted for age, sex, sleeping outside of the ward (previous 2 weeks), being treated for malaria in a previous round, use of malaria drugs (previous 2 weeks), use of ITN, complete ceiling, window screen, precipitation, and time trend. Random effects at household and TCU levels are also included.

As expected, our results were somewhat sensitive to the assumed lag length between initiation of larviciding activities and its effect on malaria transmission (Table S4). Nevertheless, the estimated effect size measures were always protective and their credible intervals did not cross the null. Results were much less sensitive to the choice of modeling process for the time trend. The effect of the choice of lag for rainfall had virtually no effect on the effect size estimate of the larviciding intervention. Finally, adding either socio-economic status, occupation of the household head, or education level of the household head did not change the odds ratio for the larviciding intervention.

**Table S4**: Sensitivity analyses of modeling assumptions and their impacts on the effect size estimate of the larviciding intervention.

| **Parameters**§ | | **Larviciding Intervention** | |
| --- | --- | --- | --- |
|  |  | **OR*** | **95% CrI**† |
|  | |  |  |
| Lag for the larviciding Intervention | |  |  |
|  | 28 days lag | **0.81** | **(0.68-0.97)** |
|  | 35 days lag | **0.79** | **(0.66-0.93)** |
|  | 45 days lag | **0.81** | **(0.69-0.96)** |
|  | 60 days lag | **0.84** | **(0.70-0.99)** |
|  |  |  |  |
| Modeling process for time trend | |  |  |
|  | 1^st^ order autoregressive | **0.79** | **(0.66-0.93)** |
|  | 1^st^ order random walk | **0.79** | **(0.67-0.94)** |
|  | 2^nd^ order random walk | **0.81** | **(0.69-0.96)** |
|  |  |  |  |
| Lag for the weekly rainfall estimates | |  |  |
|  | 7 weeks lag | **0.79** | **(0.66-0.94)** |
|  | 8 weeks lag | **0.78** | **(0.66-0.93)** |
|  | 9 weeks lag | **0.79** | **(0.67-0.94)** |
|  | 7, 8, and 9 weeks lags | **0.78** | **(0.66-0.93)** |
|  |  |  |  |
| Adding different covariates | |  |  |
|  | Quintiles of socio-economic status (asset-based) | **0.79** | **(0.66-0.94)** |
|  | Occupation of household head | **0.79** | **(0.66-0.93)** |
|  | Education level of household head | **0.78** | **(0.66-0.93)** |

Statistically significant results are bolded

§ All models are adjusted for age, sex, sleeping outside of the ward (previous 2 weeks), being treated for malaria in a previous round, use of malaria drugs (previous 2 weeks), use of ITN, complete ceiling, window screen, precipitation, and time trend. Random effects at household and TCU levels are also included.

*OR = Odds Ratio

†CrI = Credible Intervals

**References**

1. Congdon P (2006) Bayesian statistical modelling. Chichester, England ; Hoboken, NJ: John Wiley & Sons. xi, 573 p. p.

2. Besag J (1974) Spatial interaction and statistical analysis of lattice systems. Journal of the Royal Statistical Society Series B-Methodological 36: 192-236.

3. Rue H, Held L (2005) Gaussian Markov Random Fields: Theory and Applications. Boca Raton, FL: Chapman & Hall/CRC. 280 p.

4. Webster R, Pollock K, Simons T (2008) Bayesian spatial modeling of data from avian point count surveys. Journal of Agricultural Biological and Environmental Statistics 13: 121-139.

5. Sabatinelli G, Rossi P, Belli A (1986) [Dispersion of *Anopheles gambiae s.l*. in an urban zone of Ouagadougou (Burkina Faso)] Article in French. Parassitologia 28: 33-39.

6. Trape JF, Lefebvre-Zante E, Legros F, Ndiaye G, Bouganali H, et al. (1992) Vector density gradients and the epidemiology of urban malaria in Dakar, Senegal. Am J Trop Med Hyg 47: 181-189.

7. Manga L, Fondjo E, Carnevale P, Robert V (1993) Importance of low dispersion of *Anopheles gambiae* (Diptera: *Culicidae*) on malaria transmission in hilly towns in south Cameroon. J Med Entomol 30: 936-938.

8. Machault V, Gadiaga L, Vignolles C, Jarjaval F, Bouzid S, et al. (2009) Highly focused anopheline breeding sites and malaria transmission in Dakar. Malar J 8: 138.

9. Baragatti M, Fournet F, Henry MC, Assi S, Ouedraogo H, et al. (2009) Social and environmental malaria risk factors in urban areas of Ouagadougou, Burkina Faso. Malar J 8: 13.

10. Natario I, Knorr-Held L (2003) Non-parametric ecological regression and spatial variation. Biometrical Journal 45: 670-688.

11. Rue H, Martino S, Chopin N (2009) Approximate Bayesian inference for latent Gaussian models by using integrated nested Laplace approximations. Journal of the Royal Statistical Society Series B-Statistical Methodology 71: 319-392.

12. Rue H, Martino S, Lindgren F (2009) INLA: Functions which allow to perform a full Bayesian analysis of structured (geo-)additive models using Integrated Nested Laplace Approximaxion. R Package version 0.0 ed.

13. Miettinen OS (1985) Theoretical epidemiology : principles of occurrence research in medicine. New York: Wiley. xxii, 359 p. p.
